# Cells of Matter—*In Vitro* Models for Myotonic Dystrophy

**DOI:** 10.3389/fneur.2018.00361

**Published:** 2018-05-23

**Authors:** Magdalena Matloka, Arnaud F. Klein, Frédérique Rau, Denis Furling

**Affiliations:** Sorbonne Université, INSERM, Association Institut de Myologie, Centre de Recherche en Myologie, Paris, France

**Keywords:** dm1, cells, cultured, CTG repeats, models, biological, pathophysiology, human cells

## Abstract

Myotonic dystrophy type 1 (DM1 also known as Steinert disease) is a multisystemic disorder mainly characterized by myotonia, progressive muscle weakness and wasting, cognitive impairments, and cardiac defects. This autosomal dominant disease is caused by the expression of nuclear retained RNAs containing pathologic expanded CUG repeats that alter the function of RNA-binding proteins in a tissue-specific manner, leading ultimately to neuromuscular dysfunction and clinical symptoms. Although considerable knowledge has been gathered on myotonic dystrophy since its first description, the development of novel relevant disease models remains of high importance to investigate pathophysiologic mechanisms and to assess new therapeutic approaches. In addition to animal models, *in vitro* cell cultures provide a unique resource for both fundamental and translational research. This review discusses how cellular models broke ground to decipher molecular basis of DM1 and describes currently available cell models, ranging from exogenous expression of the CTG tracts to variable patients’ derived cells.

## Introduction

Myotonic dystrophies (DM) are a group of dominant disorders that are among the most prevalent neuromuscular diseases in adults (1). The main characteristics of these multisystemic diseases are myotonia, progressive muscle weakness and wasting, cardiac-conduction defects, cognitive impairments together with other endocrine dysfunctions (1). Two DM forms have been identified so far: type 1 (DM1 also called Steinert Disease) and type 2 (DM2, previously known as PROMM), which is generally less severe than DM1. Both share a similar molecular mechanism in which deleterious expansion of microsatellite repeats in non-coding regions, (CTG)n in 3′UTR of dystrophia myotonica protein kinase (*DMPK*) gene in DM1 (2–4) and (CCTG)n in intron 1 of *CNBP* gene in DM2 (5) are transcribed into expanded C/CUG-RNA that are retained in the nucleus as discrete foci. These ribonuclear foci sequester muscleblind-like (MBNL) RNA-binding proteins, resulting in their functional loss and consequently, RNA metabolism alterations (6–11). Thus, misregulation of alternative splicing events within downstream effector genes were found in striated muscles of DM1 patients and associated with clinical symptoms, such as insulin resistance, myotonia, muscle weakness, and cardiac defects (12–18).

During the past 20 years, several animal models, including mouse, fly, zebrafish, and worm have been developed to investigate DM1 pathophysiologic mechanisms. They largely contributed to the current state of the art on myotonic dystrophies, which also benefited from research performed on cell cultures. At present, more than 100 years from the first descriptions of Steinert disease, there is still a need for cellular models to decipher disease-related molecular mechanisms and evaluate therapeutic approaches before *in vivo* validation. Because DM is a multisystemic disease affecting many tissues and cell types, various cell models are required to cover all DM-associated defects. Thanks to technological progresses, we have access to new cellular models allowing more comprehensive and adequate studies. Herein, we will discuss the use of *in vitro* cell models through the advances in myotonic dystrophy research and describe the available cellular models, from exogenous expression of CTG repeats to patient’s derived cells, which were developed for the study of DM1.

## Cellular Models in DM1 Research History

In the early 1900s, Dr. Hans Steinert provided for the first time a detailed description of a neuromuscular disorder characterized by dystrophic progression with myotonia and degeneration of skeletal muscle (1). Since then, Steinert’s disease that was renamed as myotonic dystrophy type 1 or DM1 by the International Myotonic Dystrophy Consortium has been extensively investigated at both clinical and pathophysiologic level. Even before the discovery of the mutation responsible for DM1, primary cells derived from DM1 patients have been used to uncover differences in behavior or cytochemistry (19–22) to study metabolism (23–26) or to understand mechanisms leading to symptoms described in patients, like widely observed insulin resistance (27–30). However, besides learning about the clinical, physiological, and cellular manifestations of DM1, it was essential to define the molecular bases of the disease. The first breakthrough came in 1992, when the mutation responsible for DM1 was identified as an unstable CTG expansion within the 3′ non-coding region of the *DMPK* gene (2–4, 27, 31, 32). The next challenge was to understand how this expansion leads to molecular and cellular defects observed in DM1 cells. As it became striking that mutant *DMPK* mRNA was altered in DM1, the use of different cellular models provided initially confusing conclusions about its expression in the disease (33, 34). Nevertheless, the observation that the level of mutant *DMPK* mRNA decreased when the size of the repeats increased, led to the hypothesis that expanded repeats were rather impairing post-transcriptional processing of the mutant DM1 allele (35). The compelling evidence for this postulate came shortly after, when discrete ribonuclear foci were reported for the first time in DM1 fibroblasts (36). Additionally, experiments performed with patient-derived myoblasts and fibroblasts determined that mutant *DMPK* transcripts, while correctly spliced and polyadenylated, were not exported to the cytoplasm but retained in the nucleus (37, 38), causing approximately 50% reduction of the DMPK protein levels in DM1 myoblasts (39). These findings obtained from patient-derived cells gave rise to the idea of a RNA gain-of-function mechanism in DM1. This concept was proposed following the identification of a RNA-binding protein, CELF1 (also called CUG-BP) that could bind to single-stranded UG motifs and is aberrantly accumulated in the nucleus of cells derived from DM1 patients (40–43). Upregulation of CELF1 and its splicing regulatory activity have been associated with abnormal splicing of downstream targets, suggesting a trans-dominant effect of CUG repeats on RNA processing in DM1 (18, 44) which was further confirmed in cell models overexpressing exogenous CUG expanded tracts with increasing lengths (16). Finally, at the beginning of the 2000s, a second breakthrough has been reached with the identification of RNA-binding proteins that bind specifically to CUG repeats proportionally to the size of the expansion (45). These proteins belong to the MBNL family, which includes three paralogs (MBNL1, MBNL2, and MBNL3), and all of them are sequestered within the nuclear RNA foci in DM1 patient cells (46). Among their functions, MBNL proteins are splicing regulatory factors that control developmental switch between fetal and adult isoforms of many transcripts (47). Thus, titration of MBNL proteins by nuclear CUGexp-RNA results in alternative splicing misregulations of several pre-mRNAs in DM1, and some of them are associated to DM1 phenotypic features, establishing the deficiency of functional MBNLs as a central cause of the disease (12–18, 48–51).

## Cell Models Expressing Exogenous CTG Repeats

Several years after the identification of the mutation, the expression of exogenously expressed CTG tracts in cellular models was widely used as a tool to confirm the direct role of the repeats in the pathologic mechanisms of DM1. The repeats, usually inserted in the 3′UTR of a truncated *DMPK* gene commonly under the control of a CMV promoter, are transiently or stably expressed in well-characterized human or murine cell lines, such as HeLa, HEK, or C2 cells. Even if they are lacking the entire genomic context of the CTG expansion and its own specific promoter regulation, they still recapitulate several DM1-associated features like the formation of ribonuclear foci that colocalizes with MBNL proteins and the splicing defect (16, 52, 53). Thus, they provide fast and reproducible tools for informative screening readout. Up to date, several constructs containing expanded CTG repeats have been described. A construct expressing interrupted 960 CTG repeats has been used in a wide range of studies including molecular mechanism investigations and validation of therapeutic approaches (54). To ensure the stability of the expansion, the CTG tracts are interrupted with TCGA sequences every 20 repeats (16). Several similar constructs with different lengths of CTG repeats and/or promoters were developed (16, 52, 55, 56), however, the potential impact of these interruptions is not well defined yet. Additionally, constructs expressing short but pure repeats were also developed. Stable muscle cell lines expressing 200 CTG showed nuclear aggregates of mutated RNA that may cause disruptions in myogenic differentiation according to the 3′UTR-*DMPK* environments of the CTG repeats (57, 58). Overexpression of large pure repeats is more challenging due to their instability and technical issues associated with the cloning of long tracts of CTG repeats that substantially restricts their length (59, 60). However, some works partially overcame this constraint by expressing plasmids reaching 800 and 914 uninterrupted CTG repeats in the 3′UTR context of the *DMPK* gene or in inducible construct expressing GFP, respectively (10, 61–63).

*In vitro* cell models expressing exogenous CTG repeats have been widely used for small molecules screenings, therapeutic approaches, or molecular investigations (16, 54–56). However, these models may encounter some limitations associated with the level of CTG overexpression that is not under the control of the endogenous *DMPK* promoter and the absence of the complete *DMPK* genomic context that could limit their use in specific tissue or molecular mechanism studies.

## DM1 Patients’ Derived Cell Models

**Figure 1:**
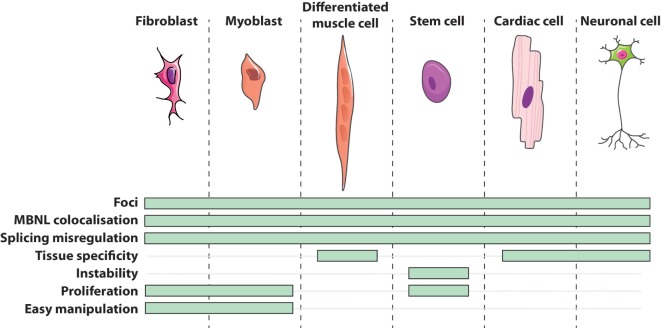
Features of DM1 cells *in vitro*.

Cells obtained directly from patients are of great utility in modeling human genetic disorders if they reproduce molecular hallmarks of the disease. Regarding DM1 patients’ derived cells, they express the whole range of mutation lengths observed in affected individuals within their natural genomic context and reproduce other canonical features of the disease (Figure 1) such as CUGexp-RNA foci that colocalize with the MBNL family members (45, 46, 64–70), alternative splicing misregulations (48, 65, 67, 70–73), and alterations of metabolic pathways (74, 75). However, considering variable parameters, like for instance samples from patients with different forms of the disease (from congenital to adult), culture conditions, or replicative senescence of primary cells, one should be aware of experimental variability between them. The generation of patient’s cell lines or stem cells with their vast reprogramming abilities represent additional tools for deciphering molecular DM1 pathogenesis but also for translational research including drugs screening (72, 76) and therapeutics development (65, 70, 77–79).

### Human Primary Cells

Currently in the DM1 field, primary cell cultures mainly consist of dermal fibroblasts and skeletal muscle cells, also called myoblasts. Isolated directly from patient’s tissue by either an enzymatic digestion of the extracellular matrix or an explant technique (small sliced pieces of biopsy from which cells grow out), primary cells potentially maintain physiological characteristics of their origin tissue environment. Primary fibroblasts are often used because of their relative accessibility from patients and their ease of manipulation in culture. On the other hand, primary myoblasts have the advantage to initiate a myogenic differentiation that results in their fusion into multinucleated cells or myotubes (Figure 1). Concomitantly, the expression of the *DMPK* gene is upregulated during the myogenic differentiation process (39) and differentiated DM1 muscle cells conserve some features found in DM1 muscles such as alternative splicing misregulation of muscle-specific transcripts (49, 78–88). Given the relative difficulty to have access to muscle biopsies of dystrophic patients, an alternative source of muscle-like cells was designed. Thus, primary fibroblasts were transduced with a viral construct expressing the key myogenic factor MYOD1 to force the expression of the myogenic program. This trans-differentiation leads to the formation of differentiated muscle-like cells expressing muscle-specific transcripts presenting similar splicing abnormalities that those found in DM1 primary myoblasts and patients’ muscles (18, 89, 90).

However, working with primary cells has also some constraints. Asides from the limited accessibility and availability of biopsies from patients, all somatic cells enter into replicative senescence after a define number of divisions that is inversely correlated with the age of the donor (91, 92). This phenomenon is even more pronounced in DM cells as their proliferative capacity is reduced when compared with age-matched control due to a premature entry into replicative senescence (81). Another difficulty, except the lengths of the repeats itself, is that primary cells could reflect the variability of the individual they are isolated from. Indeed, the age of the donor (fetal vs. adult), the tissue origin (distal vs. proximal muscle), and impairment, and the severity of the patient symptoms could influence cells behavior when grown in culture. In addition, various optimizations of cell cultures and medias or manipulation conditions may potentially lead to discrepancies between results.

### Immortalized Human Fibroblast and Myoblasts

**Figure 2:**
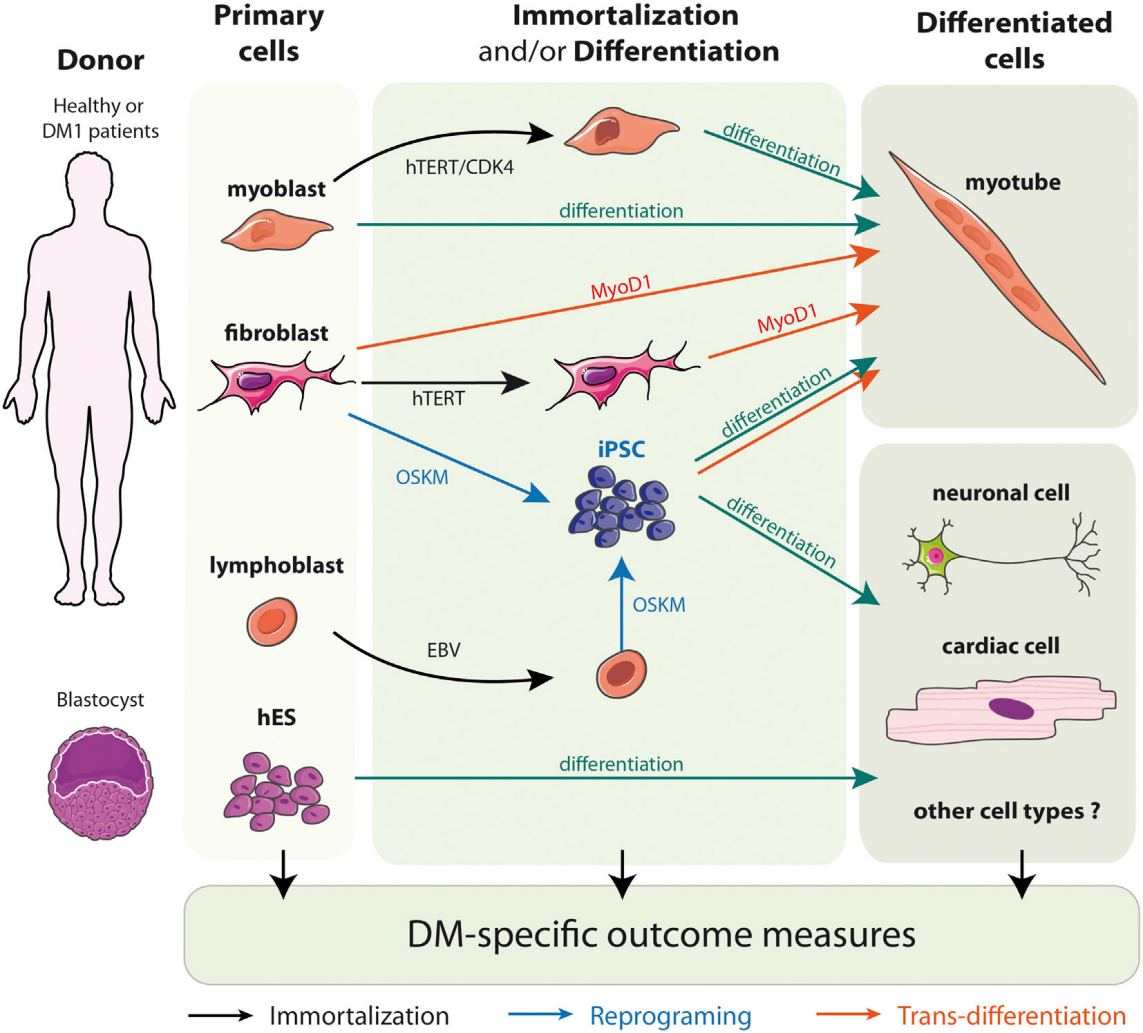
Models of DM1 patients’ derived cells.

To circumvent the limitation of replicative senescence and keep the cells in a proliferative state, immortalized cell lines from DM1 primary fibroblasts, trans-differentiated fibroblasts, and myoblasts have been established (Figure 2) (71, 76, 93–95). The immortalization process of human fibroblasts requires the stable re-expression of the human telomerase (hTERT) to prevent the excessive shortening of telomeres that triggers the entry in replicative senescence. Additional inhibition of the dominant p16 pathway by overexpressing CDK4 (the natural ligand of p16) in association, or not, with CCND1 is needed for the immortalization of human myoblasts (96–98). As a result, the immortalized DM1 cell lines display potentially unlimited number of divisions while keeping most of the tissue- and disease-specific characteristics. Furthermore, clonal selection leads to homogeneous cell cultures, which allow to provide more consistent and reproducible results. Immortalized cells due to their unlimited lifespan are of special interest when considerably large amounts of cellular material is needed, e.g., for high-throughput screenings (54, 76, 99).

Although the immortalized cell lines present a high value, it is not fully determined yet whether the immortalization process that requires viral transduction for genomic integration and stable expression of hTERT and CDK4 transgenes has any consequences on cellular behavior. Further validations and the use of different cell lines might also be needed to determine whether increasing number of divisions may alter disease- or tissues-characteristics of immortalized cells.

### Human Pluripotent Stem Cells (hPSCs)

Cultures of some primary cells types like human neuronal cells constitute a major challenge due to limited biopsies availability and delay of tissue harvesting. The difficulty to obtain material reflecting early stages of the disease process also represents considerable limitations for disease investigations (100). Fortunately, the ability to generate defined cell types from hPSCs offers a unique opportunity to study disease mechanism in a cell-specific manner. hPSCs comprising both embryonic and induced pluripotent stem cells (iPSCs), carry the potential to differentiate them into a wide spectrum of cell types *in vitro*, including the recapitulation of early human embryo development states (101–103). Therefore, they provide an attractive prospect for modeling cell-type-specific disorders.

Embryonic stem cells (hESCs) are isolated from the inner mass of the blastocyst, and can be distinguished by their remarkable long-term proliferative potential along with the ability to differentiate into practically any cell type (Figure 2) (102). Although hESCs allow the generation of diverse cell types, up to date most of the work in the DM1 field has been performed in neural stem cells. Lately, they proved to be additionally useful in identification of new pathways misregulated in the context of DM1 mutation-like disruptions in mTor signaling or defective neuritogenesis (64, 67). However, hESC research raises several ethical issues and has been a subject of controversy over the past 15 years. To obviate the concerns of hESCs, a more recent method of generating patient-specific cells has arisen. It is built on the discovery that somatic cell nuclei can be “reprogrammed” to an embryonic-like state. The whole process begins for obtaining somatic cells, e.g., fibroblasts, from the affected individuals, which are then subjected to delivery of reprogramming factor cocktails (Figure 2). Such modified cells are referred to as iPSCs (101). Most importantly, this approach can be applied to human somatic cells, offering a unique opportunity to derive patient-specific cell lines from readily available material (100, 101). Several hiPSc derived from primary fibroblasts of DM patients have already been described (65, 73, 104–107). Interestingly, the ability to reprogram DM1 immortalized lymphoblastic cells carrying 200 CTG repeats into hiPSC also opens the possibility to obtain, in the near future, hiPSC directly from patient’s blood samples rather than skin biopsies (106).

Intriguingly, regarding the pluripotent state of the cells, CTG repeats are highly unstable during both reprogramming and subsequent passages, with a more rapid expansion when the initial CTG tract is longer (108). Contradictory to what is observed *in vivo*, several reports have shown CTG repeats instability during culture of undifferentiated DM1-hPSCs but not in differentiated cells, which might be related to some epigenetic differences in these cells (73, 108, 109). In DM1 maternally derived hESC lines, hypermethylation occurs upstream of CTG repeats when repeats number exceeded 300, however, the hypermethylation observed during reprogramming of patients fibroblasts into hiPSCs is not associated with the expansion of CTG repeats (110, 111).

In the past years, a substantial progress in the culture and differentiation technologies associated with hiPSC has been done. Comparing to tissue harvesting, hiPSCs have the advantage of a nearly endless supply. They might be expanded to large quantities and stored for a future expansion or manipulation. Nevertheless, the equivalence of iPS-derived cells to mature *in vivo* cells might vary because they often do not capture the entire mature phenotype. Also, other issues such as homogenous culture of differentiated cells, chromosomal rearrangement during reprogramming and relatively high cost of hPSC maintenance, constitute additional challenges. However, this technology offers a unique opportunity to investigate specific human disease cell types such as neuronal cells or cardiomyocytes for which there are none other or highly limited biological resources.

## DM1 Cell Models as a Tool for the Development of Therapeutic Approaches

*In vitro* studies using DM1 cell models contribute also to the development of therapeutic approaches for myotonic dystrophies. Comprehensive studies have been performed in various DM1 cell models to determine and support translational potential of new strategies. Thus, different approaches aim to degrade mutant *DMPK* mRNAs have been tested in DM1 derived cells including gapmer antisense oligonucleotides (ASOs) directed against the CUGexp repeats (55, 112) or the *DMPK* transcript it-self (113) as well as shRNA (114), which have showed significant efficacy in decreasing the level of CUGexp-transcripts. In another hand, CUGexp-steric blocking approaches by using fully modified ASOs or viral-derived antisense RNA proved also to be effective in reversal disease molecular features when tested in DM1 cellular models (77, 78, 112, 115, 116). As a matter of fact, DM1 cells are not only used for therapeutic compounds validation. Indeed this tool is also utilized in screening assays allowing the identification of molecules that either interfere with the abnormal MBNL1:CUGexp interaction such as pentamidine (117, 118) or lomofungin (119), reduce the expression of mutant *DMPK* mRNAs like actinomycin D, modulate splicing changes (72) or affect the behavior of nuclear foci (120–122). Besides, reliable DM1 cell models are essential in the perspective of the recent progress made in genome engineering. TALEN and CRISPR-Cas approaches are being successfully applied in different disease cellular models giving rise to a wide range of possibilities for future therapeutic interventions (65, 70, 95, 123–125). DM1 cell models constitute, therefore, an inescapable source and a flexible platform for thorough studies and validation of disease therapeutics.

## Conclusion

Through the years, cultured cells showed to be an essential model for both fundamental and translational research on myotonic dystrophy. Despite the fact that cells do not reflect the complexity of a whole organ or body, each cellular model, from the patients’ derived cells to more artificial models overexpressing CTG expanded tracts, is suitable for different investigations. They were and are used in many studies addressing various questions related to myotonic dystrophy diseases like mutation lengths, instability, polymorphisms or tissue-specific mechanisms, molecular alterations, and effect of therapeutic approaches. Understanding all of those features paved the way to decipher molecular basics of DM1 and DM2, as both forms share common features, i.e., abnormal expansion of repeated sequences, formation of RNA-positive foci and trans-dominant effect on alternative splicing. Even though DM1 cells served as archetype for DM research, it is noteworthy that some cellular models have been also established for myotonic dystrophy type 2. Further investigations in those cells may emphasize the differences between both DM forms and promote better understanding of their pathological mechanisms. Besides, recent advances in cell-availability and -engineering have given rise to unprecedented experimental opportunities to study disease mechanisms and therapeutic strategies. Late genome engineering tools, with the particular use of emerging development of TALENs and the CRISPR–Cas9 systems, facilitate the next generation of therapeutic interventions and hold a great promise for permanent genetic corrections (65, 70, 95, 123–125). Furthermore, it opens the door for the development of isogenic cell lines providing a genetically matched “control cells.” Alternate possibilities brought by genome editing tools combined with hiPSC technologies promise the generation of novel tissue-specific cell lines opening new horizons for the development of more refined wide-ranging myotonic dystrophy cell models, which will push forward future disease investigations.

## Author Contributions

MM, AK, FR and DF wrote the review.

## Conflict of Interest Statement

The authors declare that the research was conducted in the absence of any commercial or financial relationships that could be construed as a potential conflict of interest.
